# Linoleic Acids Overproducing *Lactobacillus casei* Limits Growth, Survival, and Virulence of *Salmonella* Typhimurium and Enterohaemorrhagic *Escherichia coli*

**DOI:** 10.3389/fmicb.2018.02663

**Published:** 2018-11-01

**Authors:** Mengfei Peng, Zajeba Tabashsum, Puja Patel, Cassandra Bernhardt, Debabrata Biswas

**Affiliations:** ^1^Department of Animal and Avian Sciences, University of Maryland, College Park, College Park, MD, United States; ^2^Biological Sciences Graduate Program – Molecular and Cellular Biology Concentration, University of Maryland, College Park, College Park, MD, United States; ^3^Center for Food Safety and Security Systems, University of Maryland, College Park, College Park, MD, United States

**Keywords:** lactic acid bacteria, foodborne enteric bacterial pathogens, conjugated linoleic acid, anti-pathogenesis, anti-inflammation

## Abstract

Probiotics, particularly lactic acid bacteria, are biologic agents which limit the growth, virulence, and survival/colonization of various enteric bacterial pathogens and serve as potential alternatives to antibiotics. Mechanisms that contribute to this antimicrobial effect include producing bioactive metabolites/acids, increasing nutrient and receptor-mediated competition, and modulating gut microbiome ecology. However, these functions of common probiotic strains are limited due to the finite quantity of metabolites they produce and their total number in the gut ecosystem. Conjugated linoleic acids (CLAs), critical metabolites of *Lactobacillus*, have multiple beneficial effects on human health including anti-carcinogenesis, anti-inflammation, anti-oxidation, and anti-pathogenicity. In this study, we aim to overexpress the myosin cross-reactive antigen gene (*mcra*) in *Lactobacillus casei* (LC) to enhance the production of CLA and investigate its effectiveness against enteric bacterial pathogens, specifically *Salmonella enterica* serovar Typhimurium (ST) and enterohaemorrhagic *Escherichia coli* (EHEC). By inserting *mcra* in *L. casei*, we generated LC-CLA and found the total linoleic acid production by an individual bacterial cell was raised by 21-fold. The adherence ability of LC-CLA on human epithelial cells increased significantly and LC-CLA competitively excluded both ST and EHEC in a mixed-culture condition. Furthermore, LC-CLA significantly altered the physicochemical properties, biofilm formation abilities, interactions with host cells of both ST and EHEC, and triggered anti-inflammatory activities of host cells. These findings offer insights on applying a genetically engineered probiotic to control gut intestinal infections caused by ST and EHEC and prevent foodborne enteric illness in human.

## Introduction

Human enteric microbial infections are principally characterized by diarrhea with or without other complications/consequences, which causes approximately 4–6 million deaths annually and possesses huge economic burden worldwide (Viswanathan et al., 2009; Christou, 2011). The dominant causative agents of enteric bacterial diseases include *Salmonella*, enterohaemorrhagic *Escherichia coli* (EHEC), *Campylobacter*, *Listeria monocytogenes*, and *Shigella* (Viswanathan et al., 2009; Mor-Mur and Yuste, 2010; Forsythe, 2016; Huang et al., 2016). These enteric bacterial pathogens are typically acquired through contaminated foods and water; therefore, risk is always associated with these foodborne diseases for everyone living on this planet. The Center for Disease Control and Prevention (CDC) estimated that in the United States alone, 48 million illnesses (approximately 1 in 6 Americans), more than 128 thousand hospitalizations, and thousands of deaths are caused by foodborne infections each year (Hoffmann et al., 2012; Adams et al., 2015, 2016, 2017). The most predominant causative foodborne infectious agents, including *Salmonella* *enterica* serovar Typhimurium (ST) and EHEC, commonly colonize in farm animals’ guts, and during normal food production or processing, these pathogens often cross-contaminate meat products (Peng et al., 2014, 2016, 2018b; Salaheen et al., 2016b, 2017).

Probiotics, as bio-agents, can be considered the priority in prevention and control of foodborne bacterial pathogen-induced enteric illness (Amalaradjou and Bhunia, 2012; Hayes and Vargas, 2016; Peng and Biswas, 2017; Peng et al., 2018a). Through colonizing the host’s gastrointestinal (GI) tract, these beneficial bacteria ferment or metabolize undigested dietary components; after reaching the small and large intestine, the probiotics generate/release a tremendous treasury of secondary metabolites (byproducts), most of which are associated with multiple health benefits (Flint et al., 2012; Marcobal et al., 2013). Functional metabolites from probiotics generally include bio-active polypeptides, with antimicrobial and immune-modulatory properties, as well as vitamin B, which is essential for mammalian cells in metabolism and reproduction (Stanton et al., 2005). The major byproducts of probiotics are lipid molecules, like fatty acids especially short chain fatty acids and poly-unsaturated fatty acids with various isomers (Serini et al., 2009; Louis et al., 2014). The mixed concentration of by-produced lipid molecules in human colon is approximately 50–150 mM, and these beneficial lipid molecules are active and help modulate the host’s immune responses (Louis et al., 2014).

Among these functional fatty acids, linoleic acid (LA) is one of the most crucial beneficial metabolites produced from microbial sources, including *Bifidobacterium*, *Lactobacillus*, and *Lactococcus* (Rizos et al., 2012). The mixture of positional and geometric isomers of LA (C18:2, c9, c12), as conjugated linoleic acids (CLA), distinguishes it from other fatty acids because of its wide range of benefits on host health, including anti-carcinogenesis, anti-inflammation, and anti-pathogenicity (Lee et al., 2006; Benjamin and Spener, 2009; O’Shea et al., 2012; Yang et al., 2015). Bacteria that originate from dairy and human/animal intestines, specifically lactobacillus, including LA, *L. acidophilus*, *L*. *plantarum*, and *L*. *rhamnosus*, are known as predominant CLA producing strains (Van Nieuwenhove et al., 2011); however, their CLA productivity varies and is usually limited by multiple factors, including temperature, oxygen availability, substrate concentration, etc. (Pandit et al., 2012). A number of researchers, including our lab, are focusing on stimulating the productivities of LA and CLA from microbial sources especially probiotics both at the level of the human intestine and the industry production level (Peng and Biswas, 2017).

Through our previous research, we observed relatively intense antimicrobial activities of LA against enteric bacterial pathogens such as ST and EHEC (Peng et al., 2015c). However, the LA productivity (conversion ratio) of LC remains relatively low as 4.8%. In contrast, although *L*. *rhamnosus* possesses the highest CLA conversion rate among all active *Lactobacillus* species, it has a relatively low anti-pathogen activity (Van Nieuwenhove et al., 2011). In this study, we cloned and over-expressed the *mcra* (myosin-cross-reactive antigen) gene, encoding linoleate isomerase, from *L*. *rhamnosus* GG into LA, and aimed to examine the role of this novel probiotic in limitation and control of enteric pathogenic bacteria.

## Materials and Methods

### Bacterial Strain and Their Growth Conditions

Probiotic strains, *Lactobacillus casei* ATCC 334 (LC-WT) and *L*. *rhamnosus* GG ATCC 53103, were purchased from American Type Culture Collection (ATCC, VA, United States). *Lactobacillus* strains were grown on De Man, Rogosa and Sharpe (MRS) (EMD Chemicals Inc., Gibbstown, NJ, United States) agar at 37°C for 24 h in the presence of 5% CO_2_ (Forma^TM^ Scientific CO_2_ water jacketed incubator, Thermo Fisher Scientific, Waltham, MA, United States). Enteric bacterial pathogens *Salmonella enterica* serovar Typhimurium (ATCC 14028) (ST) and enterohemorrhagic *Escherichia coli* EDL933 (ATCC 700927) (EHEC) were grown on LB agar (EMD Chemicals Inc., Gibbstown, NJ, United States) for 18 h at 37°C under aerobic conditions (Thermo Scientific, Thermo Fisher Scientific, Waltham, MA, United States).

### Cell Lines and Culture Conditions

Human epithelium cells (INT407, ATCC CCL-6) were purchased from ATCC and cultured at standard condition (37°C, 5% CO_2_, 95% humidity) in Dulbecco’s modified Eagle medium (DMEM) supplemented with 10% FBS and 100 μg/mL gentamicin (HyClone Laboratories Inc., Logan, UT, United States). The cultured cells were seeded at approximately 2 × 10^5^ cells/mL/well into 24-well tissue culture plates (BD Falcon, Franklin Lakes, NJ, United States) to reach 80–90% confluence monolayer at standard condition for cell adhesion assay. The post-confluent INT-407 cell monolayers were rinsed with PBS and stabilized in antibiotic-free DMEM for 1 h prior to the invasion assay.

Human macrophage cell line (U937, ATCC CRL3253) was purchased from ATCC and grown at standard condition in RPMI-1640 Medium supplemented with 10% FBS and 100 μg/mL gentamicin. An aliquot of 6 mL cell suspension containing 1 × 10^6^ cells were transferred into 25 cm^2^ flask (Greiner Bio-One, Monroe, NC, United States) and cultured at standard condition for 24–30 h. After time, the cell monolayer was washed for three times with RPMI for further bacterial infection.

### Over-Expression of Myosin-Cross-Reactive Antigen Gene (*mcra*) in *L. casei* and LC-CLA Development

Plasmid pJET and *E*. *coli* DH5α were purchased from Thermo Fisher Scientific (Waltham, MA, United States), pDS132 and *E*. *coli* β2155 were donated by Dr. Fidelma Boyd (Delaware University, Newark, DE, United States), and pMSP3535 were purchased from Addgene (Cambridge, MA, United States). LC-WT and *L*. *rhamnosus* GG (ATCC 53103) were harvested from overnight culture in MRS broth, followed by three times sub-culture on MRS agar plate at 37°C for 24 h in the presence of 5% CO_2_ incubator.

**FIGURE 1:**
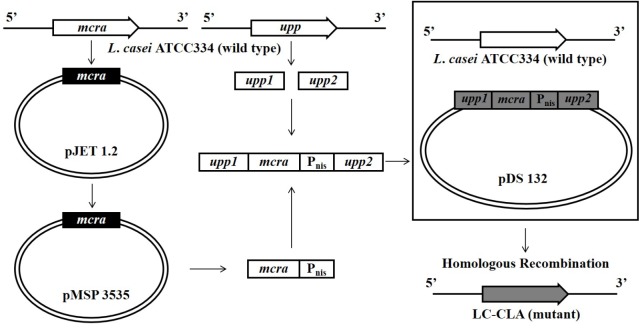
Over-expression of *mcra* in LC-WT and chromosomal recombination constructing LC-CLA.

The entire cloning design was summarized in Figure 1. Briefly, the 1750 bp *mcra* from *L*. *rhamnosus* GG was PCR amplified and ligated into pJET vector through blunt-end cloning. Aliquot of 250 μL *E*. *coli* DH5α bacterial suspension in cold 50 mM CaCl_2_ was mixed with 10 μL ligated product (pJET-*mcra*) for 10 min incubation on ice, followed by 50 s incubation at 42°C in water bath. After further 2 min incubation on ice, 250 μL LB broth was added into bacteria-plasmid mixture for 10 min incubation at room temperature followed by selection on LB agar with 100 μg/mL ampicillin for transformation. The *E*. *coli* DH5α-expressed *mcra* was double-excised from pJET-*mcra* with BamHI and XbaI and then ligated into pMSP3535 vector at 16°C overnight. Following the same condition, pMSP3535-*mcra* was further transformed into *E*. *coli* DH5α and mixed with LC-WT at ratios of 1:1, 1:5, and 1:10 (donor cells: recipient cells) for bacterial mating. The *L*. *casei*-pMSP3535 was harvested through consecutive sub-culture and selection on MRS agars containing 300 μg/mL erythromycin at 37°C under micro-aerophilic condition (Tabashsum et al., 2018).

### Removal of Antibiotic-Resistance Marker and *mcra* Chromosomal Recombination

The pMSP3535-*mcra* was isolated using Plasmid Mini Kit (Qiagen, Germantown, MD, United States). The gene sequence of *mcra* linked with transcription promoter *P*_nis_ was amplified by PCR using pMSP3535-*mcra* as the template. The upstream homologous arm *upp1* (208 bp) and downstream homologous arm *upp2* (211 bp) concatenated with Xba1 and Sac1 linkers were also PCR amplified using LC-WT genomic DNA as the template. Ligation of *upp1*-*mcra*-*upp2* was performed by PCR programmed for 40 cycles of 94°C for 30 s, 60°C for 30 s, and 72°C for 60 s. After pJET blunt-end cloning, pJET-*upp1*-*mcra*-*upp2* and pDS132 were double-digestion with Xba1 and Sac1, followed by sticky-end ligation for overnight at 16°C. The pDS132-*upp1*-*mcra*-*upp2* was then transformed into *E*. *coli* β2155 following the same method described above but with 0.3 mM DAP selection. The transformed *E*. *coli* β2155 was mixed with overnight cultured LC-WT at ratio of 1:1, 1:5, and 1:10 (donor cells: recipient cells) for bacterial mating. Aliquot of 1 mL of the mixed bacterial suspension was spread on MRS agar plate with 0.3 mM DAP, followed by 5 h incubation at 37°C under micro-aerophilic condition. The *L. casei*-pDS132 was harvested through sub-culture and selection on MRS agar with 30 μg/mL chloramphenicol. Individual bacterial colony was consecutively sub-cultured in fresh MRS broth and selected on MRS agar containing 100 μg/mL 5-fluorouracil (5-FU) for *upp1*-*mcra*-*upp2* chromosomal homologous recombination. Finally, the *mcra* chromosomal recombinant *L. casei* mutant was harvested and named it as LC-CLA.

### Co-culturing of *Lactobacillus* Strains With ST and EHEC

The survival and growth conditions of either ST or EHEC in the mixed culture with wild-type *L.* casei (LC-WT) or and mutant (LC-CLA) strains were investigated based on our previously described approach (Peng et al., 2015b). Briefly, bacterial cells from overnight agar plates were collected in 10 mL PBS using 10 μL sterile disposable loops. Each concentrated bacterial suspension was adjusted using PBS and measured by LAMBDA BIO/BIO+ spectrophotometer (PerkinElmer, Beaconsfield, United Kingdom) for adjusting the bacterial concentration to approximately 7 log CFU/mL. Aliquots of 400 μL adjusted bacterial suspension were added to sterilized test tubes containing 3.2 mL DMEM with 10% FBS and then incubated at 37°C for different time points (0, 2, 4, 8, 24, 48, and 72 h). After incubation, serial dilutions were performed in PBS, and then plated on agar plates (MRS agar for *L*. *casei*, LB agar for *S*. Typhimurium and EHEC) in triplicate, followed by incubation for 18 h at 37°C for growth. Bacterial CFUs were counted afterwards and results were expressed in unit of bacterial log CFU/mL as the average number from triplicate assays.

### Evaluation of Physicochemical Properties and Biofilm Formation of ST and EHEC

Both ST and EHEC were cultured at 37°C for 18 h and the cell surface hydrophobicity of both pathogens was determined following method previously described by Peng et al. (2015c). The interactions between bacteria cell surfaces were determined by the auto-aggregation assay according to Ahn et al. (2014) in triplicate using Multiskan microplate reader (Thermo Fisher Scientific, Waltham, MA, United States), and the enteric bacterial cell injury induced by *Lactobacillus* strains was evaluated according to the overlay method previously described by Ahn et al. (2014) in triplicate using Trypticase soy (TSA) agar and XLD- or MacConkey-overlaid TSA agar.

The bacterial biofilm formation was determined according to Salaheen et al., 2016a) with brief modifications. Both ST and EHEC were inoculated at approximately 5 × 10^5^ CFU/mL in 6-well plates (Corning, NY, United States) containing 22 mm × 22 mm glass slides and LB broth at 37°C without shaking. At 24, 48, and 72 h point, the glass slides were rinsed with PBS for five times, and bacterial cells were scrapped from glass slides followed by serially diluted for plating on LB agar.

### Scanning Electron Microscopic Analysis of Bacterial Cell Morphology

The ST and EHEC bacteria cells were harvested from overnight cultures and collected through 0.22 μm filter membranes. The bacteria cells were then fixed by submersing in 0.25% glutaraldehyde for 1 h (Kihm et al., 1994). The filter membranes were washed three times in sterile DI water followed by dehydration through sequential immersing the membranes in 10, 20, 50, 75, 90, and 100% (v/v) aqueous solutions of absolute ethanol. Filter membranes were then stored under anhydrous calcium sulfate overnight. To observe the morphology of the cells under SEM, the bacterial cells were sputter-coated with gold for Hitachi SU-70 FEG Scanning Electron Microscope (Hitachi Ltd., Japan) at an accelerating voltage of 5 kV.

### Adhesion and Invasion Assay

The cultured mammalian cell adhesion and invasion assays were carried out in triplicates following the method described previously by Peng et al. (2015b) with some modification. We used MOI = 1:100 of host cell and bacterial CFU for both ST and EHEC on INT407 cells in triplicate wells *ex vivo*. The INT407 cells grown in 24-well plate with 800 μL DMEM were pretreated with 100 μL DMEM (control), *L*. *casei* CFCSs, or 2 × 10^8^ CFUs *L*. *casei* bacterial cells, separately for 1 h, with each treatment in triplicate. A 100 μL aliquot of *S*. Typhimurium or EHEC PBS bacterial suspension with MOI = 100 (2 × 10^8^ CFUs) was inoculated into triplicate wells. Afterwards, the infected cells were incubated at standard condition for another 2 h, and then followed by three times washing with DMEM. The cell monolayers were lysed with 0.1% Triton X-100 for 15 min, serial diluted, and plated on agar plates (MRS agar for *L*. *casei*, LB agar for *S*. Typhimurium and EHEC) to estimate the adhesive bacterial CFU. To measure bacterial cell invasive activity, DMEM washed cell monolayers after 2 h bacterial infection was incubated in DMEM containing 10% FBS supplemented with 250 μg/mL gentamicin for 1 h, then followed by three times DMEM washing, Triton X-100 lysis, serial dilution, and eventually plating on agar plates mentioned above.

### Simulation of Enteric Bacterial Inflammation in Human Macrophage Cells

Enteric bacterial pathogen ST that provoke inflammation in human gut intestine was cultured on LB agar plate for 18 h and collected in PBS to be adjusted in approximately 1 × 10^9^ CFU/mL. A 100 μL aliquot of bacterial suspension, containing approximately 1 × 10^8^ CFU was inoculated into triplicate 25 cm^2^ flasks containing U937 cell monolayer (approximately 10^6^ host cells/flask). In the test flasks, 500 μL overnight (18 h) cell-free cultural supernatants (CFCSs) from *L*. *casei* (LC-WT and LC-CLA) strains in DMEM with 10% FBS were added during ST infection period. The infected monolayers were incubated for 24 h at standard condition, followed three times washing with ice-cold PBS for RNA extraction.

### Quantitative RT-PCR for Evaluation of Gene Expressions

Extraction of RNA from bacterial cells and human macrophage cell line, the cDNA synthesis, and the qRT-PCR were performed in triplicate according to the method described (Peng et al., 2017). The PCR reaction mixture containing 10 μL PerfeCTa SYBR Green Fast Mix (Quanta Biosciences, Beverly, MA, United States), 2 μL of each 100 nM primer (listed in Tables 1, 2), 2 μL of cDNA (10 ng), and 4 μL of RNase-free water was amplified using an Eco Real-Time PCR system with 30 s denaturation at 95°C, followed by 40 cycles of 95°C for 5 s, 55°C for 15 s, and 72°C for 10 s. All the relative transcription levels of target genes were estimated by comparative fold change. The *C*_T_ values of genes were normalized to the housekeeping/reference gene (listed in Tables 1, 2), and the relative expression levels of target genes were compared between control and treatment. The fold change in terms of expression of each individual target gene was calculated as ΔΔ*C*_T_ = [*C*_T_(target mRNA)-*C*_T_(reference mRNA)]_treatment_ - [*C*_T_(target mRNA)-*C*_T_(reference mRNA)]_control_ (Livak and Schmittgen, 2001). Quantitative RT-PCR was carried out in triplicate.

**Table 1:** Primers used for RT-qPCR analysis of EHEC and *S.* Typhimurium.

Bacteria	Gene	Primer Sequence (5′–3′)	Function
	*gapA*	F: ACTTCGACAAATATGCTGGC	Housekeeping gene
		R: CGGGATGATGTTCTGGGAA	
	*eaeA*	F: CCCGAATTCGGCACAAGCATAAGC	Attaching and effacing
		R: CCCGAATCCGTCTCGCCAGTATTCG	
	*espA*	F: GTTTTTCAGGCTGCGATTCT	Type III secretion protein
		R: AGTTTGGCTTTCGCATTCTT	
EHEC	*espB*	F: GCCGTTTTTGAGAGCCAGAA	Type III secretion protein
		R: AAAGAACCTAAGATCCCCA	
	*espD*	F: AAAAAGCAGCTCGAAGAACA	Type III secretion protein
		R: CCAATGGCAACAACAGCCCA	
	*ler*	F: ACTTCCAGCCTTCGTTCAGA	Locus of Enterocyte
		R: TTCTGGAACGCTTCTTTCGT	Effacement regulator
	*tir*	F: GCTTGCAGTCCATTGATCCT	Translocated intimin receptor
		R: GGGCTTCCGTGATATCTGA	
	50S ribosomal protein L5	F: GTAGTACGATGGCGAAACTGC	House keeping gene
		R: CTTCTCGACCCGAGGGACTT	
	*hilA*	F: TATCGCAGTATGCGCCCTTT	Transcriptional regulator
		R: CAAGAGAGAAGCGGGTTGGT	
	*hilC*	F: AATGGTCACAGGCTGAGGTG	Transcriptional regulator
		R: ACATCGTCGCGACTTGTGAA	
	*hilD*	F: CTCTGTGGGTACCGCCATTT	Transcriptional regulator
		R: TGCTTTCGGAGCGGTAAACT	
	*invA*	F: CGCGCTTGATGAGCTTTACC	Invasion protein
		R: CTCGTAATTCGCCGCCATTG	
	*invC*	F: GCTGACGCTTATCGCAACTG	Type III secretion system ATPase
		R: GGCGGTGCGACATCAATAAC	
	*invF*	F: TCGCCAAACGTCACGTAGAA	Transcriptional regulator
		R: CATCCCGTGTATAACCCCCG	
*S.* Typhimurium	*invG*	F: CGAATGACGCCAGCTGTTC	Invasion protein
		R: TGCGTCAGGCGTCGTAAA	
	*invH*	F: GGTGCCCCTCCCTTCCT	Invasion lipoprotein
		R: TGCGTTGGCCAGTTGCT	
	*orgA*	F: AGGCAGGGAGCCTTGCTT	Oxygen- regulated invasion protein
		R: CCCTGATGCATTGCCAAAA	
	*orgB*	F: ACCATCCCGAAACGCTTTTA	Oxygen- regulated invasion protein
		R: TTGCCCCTCAGGCTTATCG	
	*prgH*	F: TGAACGGCTGTGAGTTTCCA	Type III secretion protein
		R: GCGCATCACTCTGACCTACCA	
	*prgl*	F: GGTCTATGGAAACGGACATTGTC	Type III secretion protein
		R: CGCCGAACCAGAAAAAGC	
	*prgK*	F: GGGTGGAAATAGCGCAGATG	Type III secretion lipoprotein
		R: TCAGCTCGCGGAGACGATA	
	*sipA*	F: CGTCTTCGCCTCAGGAGAAT	Cell invasion protein
		R: TGCCGGGCTCTTTCGTT	

**Table 2:** Primers used for RT-qPCR analysis of U937 cells cytokine genes.

Gene	Primer	Sequence (5′–3′)	Function
18srRNA	Forward	ATCCCTGAAAAGTTCCAGCA	Housekeeping gene
	Reverse	CCCTCTTGGTGAGGTCAATG	
IL-1β	Forward	GCCATGGACAAGCTGAGGAAG	Inflammatory cytokine gene
	Reverse	GTGCTGATGTACCAGTTGGG	
IL-6	Forward	GAACTCCTTCTCCACAAGCG	Pro-/Anti-inflammatory cytokine gene
	Reverse	TTTTCTGCCAGTGCCTCTTT	
IL-10	Forward	AGCAGAGTGAAGACTTTCTTTC	Anti-inflammatory cytokine gene
	Reverse	CATCTCAGACAAGGCTTGG	
IL-12	Forward	AATGTTCCCATGCCTTCACC	Pro-inflammatory cytokine gene
	Reverse	CAATCTCTTCAGAAGTGCAAGGG	
IL-23	Forward	GACACATGGATCTAAGAGAAGAG	Inflammatory cytokine gene
	Reverse	AACTGACTGTTGTCCCTGAG	
TGF-β	Forward	CTTGCTGTCCTCCTCTGCAC	Anti-inflammatory cytokine gene
	Reverse	TCACTGGGGTCAGCACAGAC	
TNFα	Forward	CAGAGGGAAGAGTTCCCCAG	Inflammatory cytokine gene
	Reverse	CCTTGGTCTGGTAGGAGACG	
CXCL-8	Forward	CTGCGCCAACACAGAAATTA	Inflammatory chemokine gene
	Reverse	ATTGCATCTGGCAACCCTAC	

### Statistical Analysis

All the data were analyzed by the Statistical Analysis System software. The one-way analysis of variance followed by Tukey’s test was applied to determine the significant differences of bacterial counts, physicochemical values, and virulent gene expression levels among the control and treatments based on a significant level of 0.05.

## Results

### Phenotypical Characterization of LC-CLA

**FIGURE 2:**
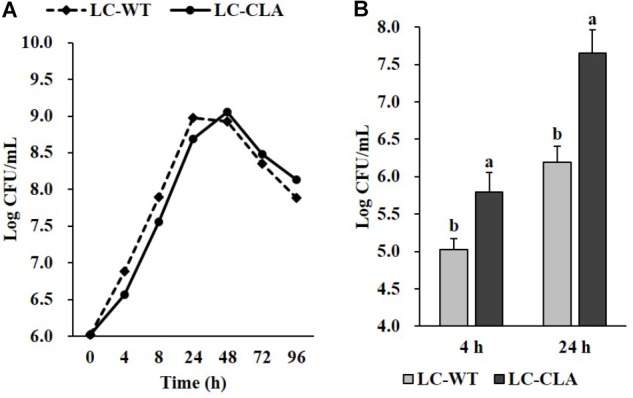
Phenotypic characterization of LC-CLA. The comparative growth of 96 h **(A)** and *ex vivo* adherence on human epithelial cells at 4 and 24 h **(B)** were examined in triplicate and compared between LC-WT and LC-CLA. Bars indicate average ± standard deviation from parallel trials. Letters (‘a’ and ‘b’) indicate significantly different between LC-WT and LC-CLA on host cell adherence over three biological repetitions at *p* < 0.05.

In comparison with LC-WT, LC-CLA maintained their *in vivo* growth/survival rate during exponential, stationary and death phases up to 96 h (Figure 2A) and remarkably (*p* < 0.05) improved their host cell adhesion ability onto human epithelial (INT-407) cells *ex vivo* (Figure 2B). The INT-407 cell-attached amount of LC-CLA was found to be significantly higher at 4 and 24 h of incubation comparing with LC-WT. In addition, the genetically engineered probiotic strain LC-CLA induced significant (*p* < 0.05) up-regulation on *mcra* (linoleate isomerase gene) mRNA level expression identified by qPCR; with HPLC-MS/MS analysis, we also detected fold increment in relative total linoleic acids per 1 mL overnight cultural supernatant as well as even higher fold boost in relative total linoleic acids per bacterial cell (Table 3).

**Table 3:** Relative expression level of *mcra* and relative production rate of linoleic acids in fold-change^∗^.

Strain	Genotype	*mcra* mRNA expression^1^	RTLA^2^ per mL supernatant	RTLA^2^ per bacterial cell
LC-WT	Wild type	1.00	1.00	1.00
LC-CLA	*mcra* over-expressed	7.15 ± 1.76	4.48 ± 0.59	21.06 ± 1.33

*^∗^Gene expression level and metabolite production rate is standardized with LC-WT. ^1^mcra expression fold change was calculated based on 16S rRNA as reference gene. ^2^Relative Total Linoleic Acids based on HPLC-MS/MS analysis.*

### Competitive Exclusion of Enteric Bacterial Pathogens, ST and EHEC

**FIGURE 3:**
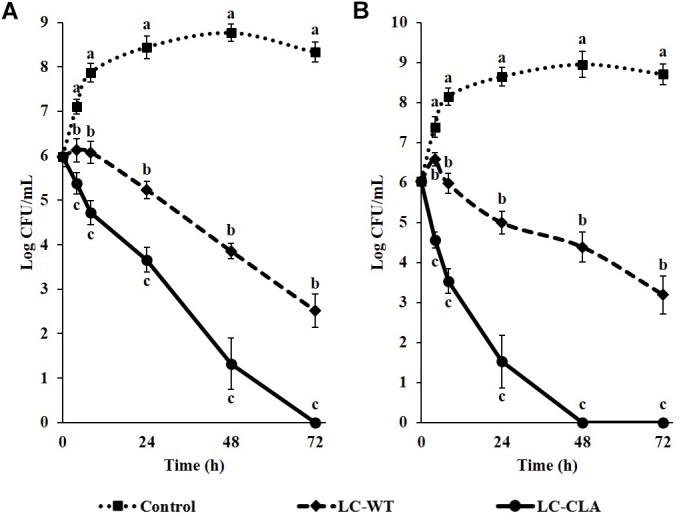
Competitive exclusion of enteric bacterial pathogens by either LC-WT or LC-CLA. Comparative growth of ST **(A)** and EHEC **(B)** in single-culture or mix-culture with LC-WT or LC-CLA over 72 h was evaluated in triplicate. Bars indicate average ± standard deviation from parallel trials. Different letters (‘a’ through ‘c’) at single time point are significantly different in growth of ST or EHEC among control and treatments over three biological repetitions at *p* < 0.05.

Probiotic *Lactobacillus* (LC-WT or LC-CLA) strains and enteric bacterial pathogens (ST or EHEC) were grown in mixed-cultured condition *in vitro* to investigate their competitive survival ability through competition between them in both short (4 and 8 h) and long (up to 72 h) period of time. The competitive inhibitory abilities of both LC-WT and LC-CLA against ST or EHEC were shown in Figure 3. Specifically, LC-CLA rapidly started to phase out both enteric bacterial pathogens with significantly (*p* < 0.01) higher loads of ST and EHEC reduction during the first 8 h incubation comparing with LC-WT. Overall, LC-CLA competitively exclude ST at 72 h and EHEC at 48 h.

### Metabolites From LC-CLA in Combating Against Enteric Bacterial Pathogens

**FIGURE 4:**
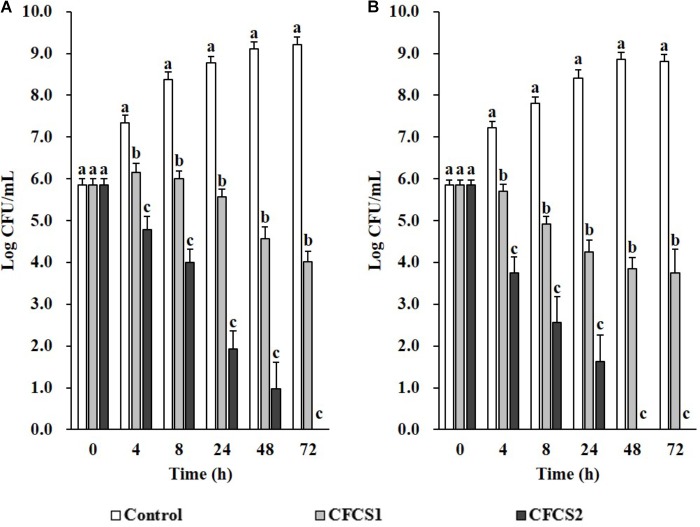
Antimicrobial activities of LC-WT and LC-CLA metabolites on EHEC and ST growth and survival abilities. Inhibitory effects of CFCSs from LC-WT (CFCS1) or LC-CLA (CFCS2) were detected on growth of ST **(A)** and EHEC **(B)** over 72 h from triplicate biological experiments. Bars indicate average ± standard deviation from parallel trials. Different letters (‘a’ through ‘c’) at single time point are significantly different in growth of ST or EHEC among control and treatments over three biological repetitions at *p* < 0.05.

Overnight CFCSs from both LC-WT (CFCS1) and LC-CLA (CFCS2), in terms of initial inoculum of 10^6^ CFU/mL overnight probiotic culture, were collected for examination the antimicrobial activities of their secreted byproducts. Comparing with negative control (only medium), both CFCSs from LC-WT and LC-CLA strains inhibited the growth of both pathogens, ST and EHEC, however, CFCS2 from LC-CLA showed more intensive effects (Figure 4). To be specific, CFCS2 reduced notably (*p* < 0.01) higher loads of ST and EHEC in the early stage at 4 and 8 h compared with CFCS1. The inhibitory activity of CFCS1 was attenuated after 24 h, whereas metabolites from LC-CLA exhibited a stable antimicrobial activity after 24 h, which ruled out all survival ST at 72 h and EHEC at 48 h.

### Alterations in Physicochemical and Morphological Properties of ST and EHEC

The produced metabolites from both LC-WT and LC-CLA in CFCSs alter multiple physicochemical properties of both pathogens, ST and EHEC (Table 4). For example, CFCS1 decreased bacterial surface hydrophobicity of ST and EHEC, whereas CFCS2 exhibited more profound effectiveness in significantly lowering hydrophobicity of both pathogens (Table 4). Following the same trend, metabolites produced by LC-CLA in CFCS2 significantly reduced bacterial auto-aggregation activities of both ST and EHEC compared with metabolites from LC-WT. Similarly, we found that CFCS2 could intensify the effect of bacterial cell wall disruption of both ST and EHEC.

**Table 4:** Physicochemical properties of ST and EHEC with CFCS treatments.

Treatment	Hydrophobicity (%)		Auto-aggregation (%)		Injured bacterial cells (%)	
	ST	EHEC	ST	EHEC	ST	EHEC
Control	18.01 ± 0.32^a∗^	14.56 ± 0.83^a^	14.72 ± 0.41^a^	6.79 ± 0.91^a^	19.80 ± 1.79^c^	16.98 ± 4.18^c^
LC-WT	10.85 ± 0.35^b^	11.39 ± 0.77^b^	8.65 ± 0.32^b^	5.24 ± 0.29^a^	30.92 ± 5.55^b^	38.28 ± 2.74^b^
LC-CLA	6.32 ± 0.43^c^	4.35 ± 0.65^c^	5.11 ± 0.41^c^	3.02 ± 0.55^b^	42.84 ± 2.64^a^	50.64 ± 4.15^a^

*^∗^Means with different letters (a–c) in individual column are significantly different at p < 0.05 between control and treatments.*

**FIGURE 5:**
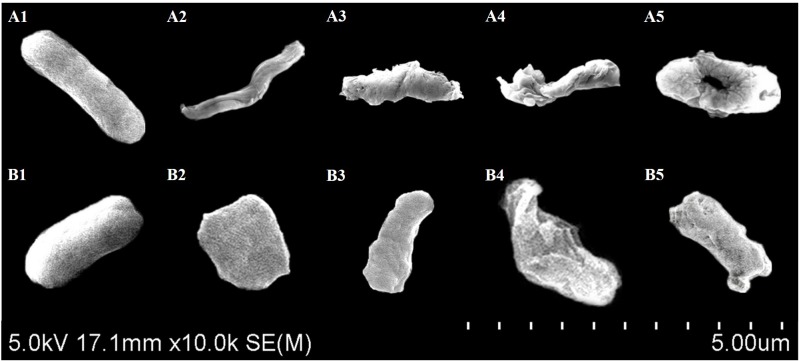
Scanning electron microscopy for bacterial cell morphology. Comparable ST **(A)** and EHEC **(B)** morphology was observed and compared between control **(A1,B1)**, CFCS1 treatment **(A2,A3,B2,B3)**, and CFCS2 treatment **(A4,A5,B4,B5)**.

The bacterial cell morphology of ST/EHEC treated with CFCSs collected from LC-WT (CFCS1) or LC-CLA (CFCS2) was examined by scanning electron microscopy (Figure 5). Comparable ST and EHEC cells were observed for morphological changes including elongation, shrinkage, and swelling during the treatment with CFCS1 (Figures 5A2,A3,B2,B3). Much more pronounced alterations in the bacterial cell morphology were also observed when the cells were treated with CFCS2, for example, enormous outer membrane disruption and immense bacterial perforation (Figures 5A4,A5,B4,B5).

### Effect on Biofilm Formation by ST and EHEC

**FIGURE 6:**
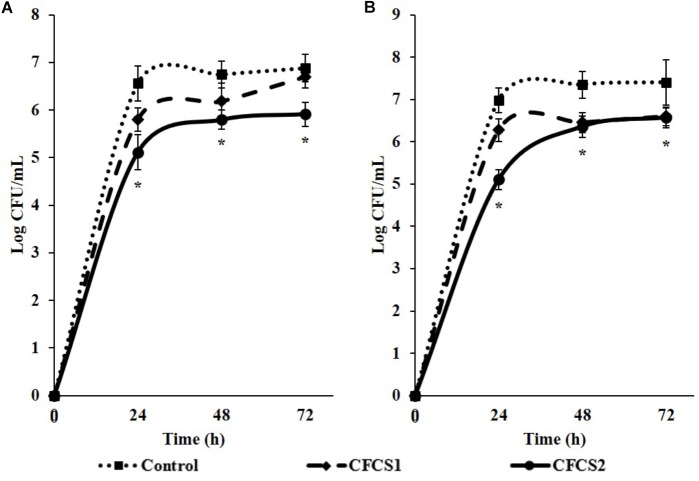
Reduction of EHEC and ST biofilm formation in the presence of either LC-WT or LC-CLA or CFCSs collected from LC-WT and LC-CLA. Comparative biofilm formation of ST **(A)** and EHEC **(B)** under pressure of CFCS from either LC-WT (CFCS1) or LC-CLA (CFCS2) over 72 h was investigated in triplicate. Bars indicate average ± standard deviation from parallel trials. Asterisks (^∗^) at single time point are significantly different in biofilm formation of ST or EHEC among control and treatments over three biological repetitions at *p* < 0.05.

The biofilm formation abilities of ST and EHEC in absence or presence of CFCSs from both LC-WT and LC-CLA are showed in Figure 6. At 24, 48, and 72 h incubation under the inhibitory pressure of LC-CLA secreted metabolites in CFCS2, the biofilm formation of ST was significantly (*p* < 0.05) suppressed. Whereas CFCS1 from LC-WT exhibited less inhibitory effects and failed to decrease the ability of ST to form a biofilm significantly after 72 h of incubation. The biofilm formation ability of EHEC was also significantly (*p* < 0.05) restrained at 24 h treatment with CFCS2 from LC-CLA. At 48 and 72 h, both CFCS1 and CFCS2 exhibited significant reduction on EHEC biofilm formation.

### Disruption on Host Cells-ST/EHEC Interactions

**FIGURE 7:**
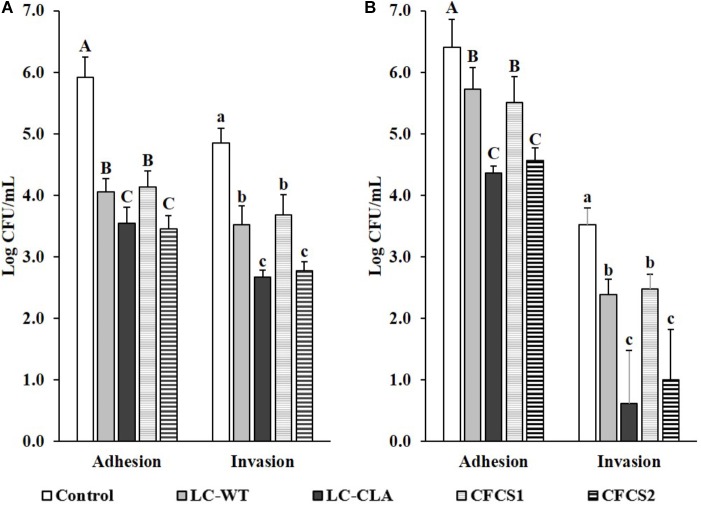
Effect of LC-CLA in interfering with enteric bacterial pathogen-cell interactions. Human epithelial cell adhesive and invasive activities of ST **(A)** and EHEC **(B)** with pre-treatment of either *L*. *casei* or CFCSs from *L*. *casei* strains were examined in triplicate. A constant MOI = 100 was applied in each sub-figure. Bars indicate average ± standard deviation from parallel trials. Different letters ‘A’–‘C’ and ‘a’–‘c’ within each bacterial pathogen are significantly different among control and treatments for cell bacterial adhesion and invasion separately over three biological repetitions at *p* < 0.05.

The host cell-ST or -EHEC interactions were evaluated based on their adhesion to and invasion into human epithelial (INT-407) cells (Figure 7). With pre-treated of LC-WT, the cell adhesive and invasive abilities of ST were significantly (*p* < 0.05) reduced. In the same investigation, host cells pretreated with LC-WT also decreased the adherence abilities of EHEC, but more effective performance was observed when INT-407 cells were allowed to pre-colonize with LC-CLA. The adhesive and invasive activities of ST were suppressed by 99.58 and 99.34% separately, by LC-CLA. Similarly, the pre-colonized LC-CLA also reduced EHEC host cell adhesion capabilities by 99.10.

Correspondingly, the pre-treatments of ST and EHEC with CFCSs collected from both LC-WT and LC-CLA displayed significant effects on their interactions/infections with INT-407 cells. Specifically, metabolites in CFCS collected from LC-WT, CFCS1 restricted the adherence activities of both ST and EHEC as well as invasive activity of ST on INT-407 cells. Whereas, CFCS2, collected from LC-CLA, altered the interaction between INT-407 cells and ST/EHEC intensively (*p* < 0.01) by decreasing 99.66% ST and 98.53 EHEC adhesion, respectively. In the same experiment, CFCS2 reduced the invasion ability of ST by 99.15% into INT-407 cells, respectively.

### Down-Regulation on Expression of Bacterial Virulence Genes by CFCSs

**FIGURE 8:**
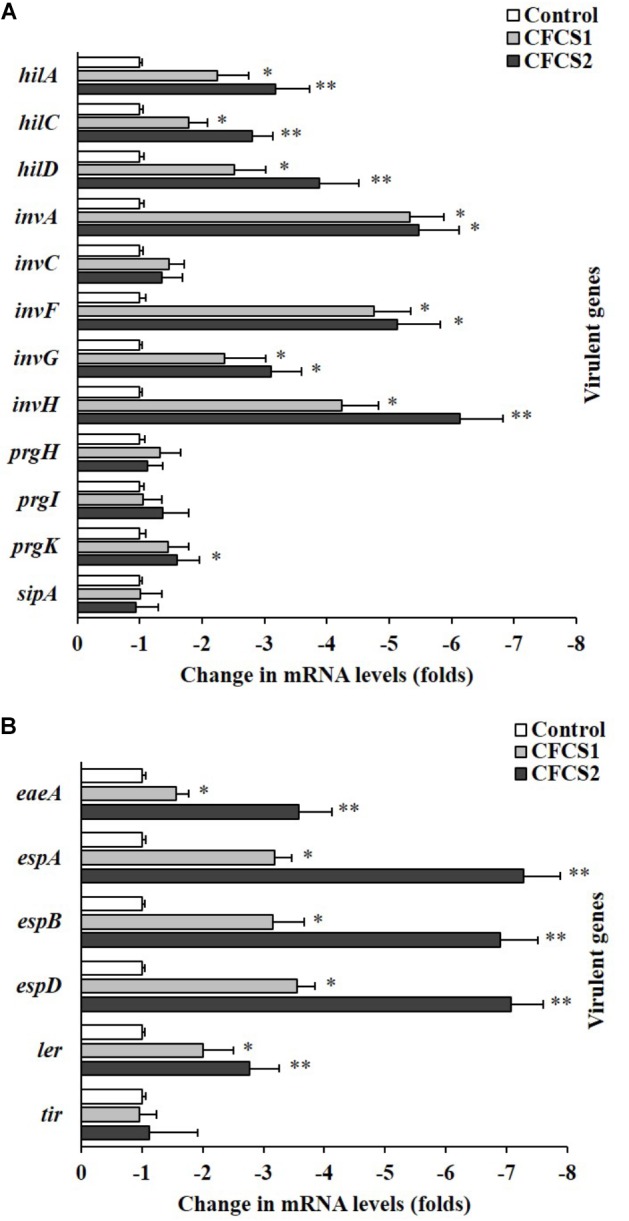
Role of LC-CLA in suppression of EHEC and ST virulence genes. The relative expression of T3SS-related virulence genes from ST **(A)** and EHEC **(B)** under pressure of CFCSs from *L*. *casei* strains was investigated in triplicate. The relative transcription levels are in the form of comparative fold change with control being 1.0. Bars indicate average ± standard deviation from parallel trials. Asterisks ‘^∗^’ and ‘^∗∗^’ indicate the significant difference in each individual virulence gene expression among control and treatments over three biological repetitions at *p* < 0.05 and *p* < 0.01 separately.

The relative expression levels of multiple ST/EHEC virulence genes were found to be significantly (*p* < 0.05) down-regulated with CFCSs from both LC-WT and LC-CLA based on qPCR analysis, among which, the suppressive effects from CFCS2 were detected to be more intensive than CFCS1 (Figure 8). For ST, CFCS2 collected from LC-CLA notably (*p* < 0.01) down-regulated the expression of transcriptional regulator genes *hilA*, *hilC*, *hilD*, and *invF* by various fold. Similarly, the expression levels of effector genes *invA*, *invG*, *invH*, and *prgK* were also significantly (*p* < 0.01) suppressed by CFCS2. Whereas, insignificant fold changes were detected in relative expression levels of *invC*, *prgH*, *prgI*, and *sipA* when the cells were treated with either CFCS1 or CFCS2. For EHEC, eight virulence genes were investigated in this study, among which only effector gene *tir* kept conservative under the pressure of both CFCSs treatment. CFCS2 effectively (*p* < 0.01) down-regulated the expression levels of regulator gene *ler* as well as other effector genes including *eaeA*, *espA*, *espB*, and *espD*.

### Anti-inflammatory Effects of LC-CLA

**FIGURE 9:**
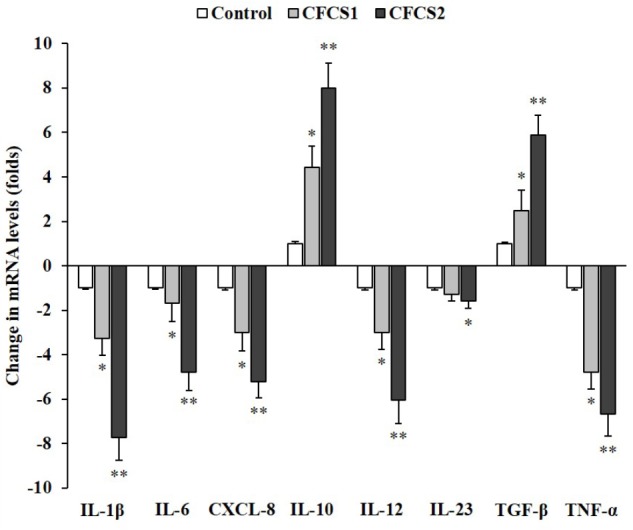
Anti-inflammatory effects of LC-CLA on human macrophage cells. The relative expression of ST-induced macrophage (anti-)inflammatory cytokine genes with treatment of CFCSs from *L*. *casei* strains was investigated in triplicate. The relative transcription levels are in the form of comparative fold change with control being 1.0. Bars indicate average ± standard deviation from parallel trials. Asterisks ‘^∗^’ and ‘^∗∗^’ indicate the significant difference in each individual cytokine gene expression among control and treatments over three biological repetitions at *p* < 0.05 and *p* < 0.01 separately.

Metabolites secreted by both *Lactobacillus* (LC-WT and LC-CLA) strains managed to induce anti-inflammatory effects on ST-induced human macrophage (U937) cells by down-regulating pro-inflammatory cytokine genes and up-regulating anti-inflammatory cytokine genes (Figure 9). In detail, CFCS1 collected from LC-WT suppressed the expression levels of IL-1β, CXCL-8 (IL-8), IL-12, and TNF-α genes by 3.3-, 3.0-, 3.0-, and 4.8-fold, respectively, and at the same experiment, it raised the expression levels of IL-10 and TGF-β genes by 4.4- and 2.5-fold, respectively. Whereas, negligible differences in fold change were observed on IL-6 and IL-23 genes expression. On the other side, CFCS2 containing metabolites released from LC-CLA impressively amplified the anti-inflammatory activities, by which relative expression levels of pro-inflammatory cytokine IL-1β, IL-8, IL-12, IL-23, and TNF-α genes were all significantly (*p* < 0.01) down-regulated by 7.7-, 5.2-, 6.0-, 1.6-, and 6.7-fold, respectively; whereas relative expression levels of anti-inflammatory cytokine IL-10 and TGF-β genes were significantly (*p* < 0.01) up-regulated by 8.0- and 5.9-fold.

## Discussion

Probiotics, prebiotics, or a combination of the two, referred to as synbiotics, have emerged as a promising alternative treatment for enteric bacterial infections (Vyas and Ranganathan, 2012; Hardy et al., 2013; Pandey et al., 2015; Peng et al., 2015c; Salaheen et al., 2015). To improve and maintain the host’s gut health, the beneficial effects of probiotics depend largely upon the total quantity and type of functional metabolites they can produce. In our recent studies, we found several prebiotic-like components in cocoa and peanuts facilitated *L*. *casei* in producing more linoleic acids and outcompeting major foodborne bacterial pathogens, including ST and EHEC (Salaheen et al., 2014; Peng et al., 2015a,b). Based on these findings, we have overexpressed the *mcra* encoding Linoleate isomerase in LC-WT to verify the ability of the genetically modified strain, LC-CLA, in combating enteric bacterial infection *ex vivo* based on the cell culture model.

As discussed in previous studies, the myosin-cross-reactive antigens, which are present across a wide range of taxa, including *Lactobacillus*, not only take responsibility in linoleic acid construction and isomerization (Kishino et al., 2011; O’connell et al., 2013; Yang et al., 2014), but also have been revealed to contribute in bacterial stress-tolerance, blood-survival, and host cell interactions (O’Flaherty and Klaenhammer, 2010; Volkov et al., 2010; Chen et al., 2016). In this study, accordingly, in comparison to LC-WT, the *mcra* overexpressed LC-CLA was found with prominently higher production of total linoleic acids, fitter growth patterns, though not statistically significant, and remarkably improved epithelial adhesion *ex vivo* especially on INT-407 cells.

Though they assist in the development of healthy gut microbiota and the maintenance of cardiovascular health, prebiotic or prebiotic-like components, contain functional foods such as peanuts and cocoa. Therefore these symbiotic combinations are not entirely ideal for antimicrobial use in long term application or in specific populations due the cost of these foods, their potential to induce allergic reactions, the ability of beneficial and pathogenic microbes to use them as an uncontrolled source of nutrients, and their limited bio-availability (Hasler, 2002; Badrie et al., 2015; Feeney et al., 2016). Therefore, the genetically engineered probiotic in our research, being self-sufficient, stands out in supply of increased bio-active byproducts devoid of any prebiotic.

As previously reported by Peng et al. (2015c), *Lactobacillus*, by releasing antimicrobial components like organic acids, hydrogen peroxide, and poly-peptides, outcompete pathogenic bacteria in a time-dependent manner. In this study, LC-CLA exhibited even stronger effects against ST and EHEC than by LC-WT in mix-culture competitive exclusion, and the CFCS2 collected from LC-CLA also showed an extensive growth inhibition effect on both pathogens, through inducing bacterial cell membrane damage. The outcomes are also supported by the previous findings on anti-pathogenic activities in CLA (Hontecillas et al., 2002; Bhattacharya et al., 2006; Meraz-Torres and Hernandez-Sanchez, 2012). Furthermore, we also surprisingly observed that due to over-expression of *mcra* in LC, LC-CLA induced significant alterations on several physiochemical properties of ST/EHEC, including surface hydrophobicity, auto-aggregation, bacterial cell morphology, and biofilm formation. The over-produced LA in LC might have induced these changes since they were suggested to interact with cytoplasmic membrane of bacterial pathogens and further disrupt phospholipid or extracellular polysaccharides (Peng and Biswas, 2017), both of which are crucial factors for bacterial physicochemical properties as well as biofilm formation (Vu et al., 2009; Renner and Weibel, 2011).

Specific virulence genes of ST/EHEC involved in Type-3 secretion (T3SS) were significantly down-regulated in the presence of the secreted metabolites in CFCS2 collected from LC-CLA. These genes include invasion regulator genes and effector genes, especially *eaeA*, that functions in EHEC A/E and *invH* encoding ST invasion lipoprotein. In fact, several research groups have also previously reported the dose-dependent activities of poly-unsaturated fatty acids in regulation of *Salmonella* and *E*. *coli* (Cardenal-Muñoz and Ramos-Morales, 2011; Nakamura et al., 2012); however, the conclusion remains to be ambiguous and bears little correlation with bacterial infections (Peng and Biswas, 2017). The repressed virulence genes and the disrupted bacterial physicochemical properties of ST and EHEC by LC-CLA served as identical indicators for the attachment of pathogens on host cells. It further supported the *ex vivo* reduction of ST/EHEC-host cell interactions excluding the negligible toxic effect of gentamycin on bacteria (Peng et al., 2015c). Through competitively occupying INT-407 cell surface receptor-like molecules (Bernet et al., 1994; Matsuo et al., 2012; Peng et al., 2015c) and enhancing the regulation of these two bacterial pathogens via the increased production of linoleic acids (Belury, 2002; Hontecillas et al., 2002; Yang et al., 2017), LC-CLA stands out with strong inhibitory actions against enteric bacterial pathogens. Though 1 h probiotic pre-occupation and 2 h pathogenic infection was investigated in this study, further research targeting up to 72 h ST/EHEC infections could be favorable in revealing the long-term preventive effects of LC-CLA.

Finally, extensive anti-inflammatory effects of LC-CLA were presented *ex vivo* on human macrophage cells. In accordance with previous studies on linoleic acids (Albers et al., 2003; Akahoshi et al., 2004; Tricon et al., 2004), we also detected a reduction in levels of pro-inflammatory cytokines/chemokines including TNF-α, IL-1β, IL-6, CXCL-8, and IL-12 in this study. Moreover, we identified the up-regulation of anti-inflammatory cytokine IL-10 and TGF-β genes as well, the two cytokines of which were believed to induce inhibition on *T*_h_ cells activation (Gorelik and Flavell, 2002; Gorelik et al., 2002; Hsieh et al., 2012). The activated macrophage cells bearing bacterial pathogen challenges normally produce and release IL-12 for activation of *T*_h_1 cells and further induces INF-γ, TNF-α, and IL-12 production (Romagnani, 1999; Dong and Flavell, 2001; Bassaganya-Riera et al., 2003; Kidd, 2003), which explained the significantly elevated expressions of TNF-α and IL-12 genes with ST infections. LC-CLA in secreting auxiliary amounts of CLA, ameliorated the ST infection-induced gut inflammatory responses by suppressing *T*_h_1 cells through reducing IL-12 and pathogenic *T*_h_17 cells through reducing IL-1β (Acosta-Rodriguez et al., 2007; Monteleone et al., 2009; Cosmi et al., 2014). Most importantly, the anti-inflammatory activities of linoleic acids have not been documented to impair any gut immunity against enteric bacterial pathogen infections (Turnock et al., 2001; Peng and Biswas, 2017).

## Conclusion

Findings from this study herald a new era, wherein non-traditional preventive strategies through using functional probiotics could become applicable in defense against enteric bacterial pathogens specifically *Salmonella* and pathogenic *E. coli*, regardless of altering the normal gut microbiota. LC-CLA with *mcra* gene over-expression managed to adhere efficiently on human epithelial cells and secret larger amounts of linoleic acids. By this pathway for combating ST and EHEC infections, the effective probiotic strain competitively excluded their growth *in vitro*, altered their physicochemical properties, as well as biofilm formation abilities, reduced their interactions to host cells *ex vivo*, and attenuated the host cell inflammatory process induced by enteric bacterial pathogens. The development and implementation of such novel, cost-effective, and simple-to-use genetically engineered probiotics, independent of prebiotics or prebiotic-like functional food ingredients, is promising to open a new avenue in prevention and treatment of *Salmonella* and pathogenic *E. coli* provoked GI infections and in improving gut health where antibiotic therapy could be limited, and helpful in avoiding negative consequences of antibiotic therapy.

## Author Contributions

MP designed the work, conducted experiments, interpreted and analyzed data, ensure the integrity of the work, and drafted and revised the manuscript. ZT performed the experiments and ensured the accuracy of the work. PP and CB conducted part of the experiments and acquired data. DB contributed to the conception and design of the research, and ensured both the accuracy and integrity of the work, and critically revised and approved the final manuscript for submission and publication.

## Conflict of Interest Statement

The authors declare that the research was conducted in the absence of any commercial or financial relationships that could be construed as a potential conflict of interest.

## References

[B1] Acosta-Rodriguez E. V. Napolitani G. Lanzavecchia A. Sallusto F. (2007). Interleukins 1β and 6 but not transforming growth factor-β are essential for the differentiation of interleukin 17-producing human T helper cells. *Nat. Immunol.* 8 942–949. 10.1038/ni1496 17676045

[B2] Adams D. Fullerton K. Jajosky R. Sharp P. Onweh D. Schley A. (2015). Summary of notifiable infectious diseases and conditions - United States, 2013. *MMWR Morb. Mortal. Wkly. Rep.* 62 1–122. 10.15585/mmwr.mm6253a1 26492038

[B3] Adams D. Fullerton K. Jajosky R. Sharp P. Onweh D. Schley A. (2017). Summary of Notifiable Infectious Diseases and Conditions - United States, 2015. *MMWR Morb. Mortal Wkly. Rep.* 62 1–122. 10.15585/mmwr.mm6253a1 26492038

[B4] Adams D. A. Thomas K. R. Jajosky R. A. Foster L. Sharp P. Onweh D. H. (2016). Summary of notifiable infectious diseases and conditions — United States, 2014. *MMWR. Morb. Mortal. Wkly. Rep.* 63 1–152. 10.15585/mmwr.mm6354a1 27736829

[B5] Ahn J. Almario J. A. Salaheen S. Biswas D. (2014). Physicochemical, Mechanical, and ##sogenic and P22-Lysogenic *Salmonella Typhimurium* Treated with citrus oil. *J. Food Prot.* 77 758–764. 10.4315/0362-028X.JFP-13-449 24780330

[B6] Akahoshi A. Koba K. Ichinose F. Kaneko M. Shimoda A. Nonaka K. (2004). Dietary protein modulates the effect of CLA on lipid metabolism in rats. *Lipids* 39 25–30. 10.1007/s11745-004-1197-3 15055231

[B7] Albers R. van der Wielen R. P. Brink E. J. Hendriks H. F. Dorovska-Taran V. N. Mohede I. C. (2003). Effects of cis-9, trans-11 and trans-10, cis-12 conjugated linoleic acid (CLA) isomers on immune function in healthy men. *Eur. J. Clin. Nutr.* 57 595–603. 10.1038/sj.ejcn.1601585 12700622

[B8] Amalaradjou M. A. Bhunia A. K. (2012). Modern approaches in probiotics research to control foodborne pathogens. *Adv. Food Nutr. Res.* 67 185–239. 10.1016/B978-0-12-394598-3.00005-8 23034117 PMC7150249

[B9] Badrie N. Bekele F. Sikora E. Sikora M. (2015). Cocoa agronomy. quality, nutritional, and health aspects. *Crit. Rev. Food Sci. Nutr.* 55 620–659. 10.1080/10408398.2012.669428 24915358

[B10] Bassaganya-Riera J. Pogranichniy R. M. Jobgen S. C. Halbur P. G. Yoon K. O’Shea M. (2003). Conjugated linoleic acid ameliorates viral infectivity in a pig model of virally induced immunosuppression. *J. Nutr.* 133 3204–3214. 14519812 10.1093/jn/133.10.3204

[B11] Belury M. A. (2002). Dietary conjugated linoleic acid in health: physiological effects and mechanisms of action. *Annu. Rev. Nutr.* 22 505–531. 10.1146/annurev.nutr.22.021302.121842 12055356

[B12] Benjamin S. Spener F. (2009). Conjugated linoleic acids as functional food: an insight into their health benefits. *Nutr. Metab.* 6:36. 10.1186/1743-7075-6-36 19761624 PMC2754987

[B13] Bernet M. F. Brassart D. Neeser J. R. Servin A. L. (1994). Lactobacillus acidophilus LA 1 binds to cultured human intestinal cell lines and inhibits cell attachment and cell invasion by enterovirulent bacteria. *Gut* 35 483–489. 10.1136/gut.35.4.483 8174985 PMC1374796

[B14] Bhattacharya A. Banu J. Rahman M. Causey J. Fernandes G. (2006). Biological effects of conjugated linoleic acids in health and disease. *J. Nutr. Biochem.* 17 789–810. 10.1016/j.jnutbio.2006.02.009 16650752

[B15] Cardenal-Muñoz E. Ramos-Morales F. (2011). Analysis of the expression, secretion and translocation of the *Salmonella enterica* type III secretion system effector SteA. *PLoS One* 6:e26930. 10.1371/journal.pone.0026930 22046414 PMC3203157

[B16] Chen Y. Y. Liang N. Y. Curtis J. M. Gänzle M. G. (2016). Characterization of linoleate 10-hydratase of *Lactobacillus plantarum* and novel antifungal metabolites. *Front. Microbiol* 7:1561. 10.3389/fmicb.2016.01561 27757104 PMC5047880

[B17] Christou L. (2011). The global burden of bacterial and viral zoonotic infections. *Clin. Microbiol. Infect.* 17 326–330. 10.1111/j.1469-0691.2010.03441.x 21129102 PMC7129620

[B18] Cosmi L. Maggi L. Santarlasci V. Liotta F. Annunziato F. (2014). T helper cells plasticity in inflammatio n. *Cytometry A* 85 36–42. 10.1002/cyto.a.22348 24009159

[B19] Dong C. Flavell R. A. (2001). Th1 and Th2 cells. *Curr. Opin. Hematol.* 8 47–51. 10.1097/00062752-200101000-00009 11138626

[B20] Feeney M. Du Toit G. Roberts G. Sayre P. H. Lawson K. Bahnson H. T. (2016). Impact of peanut consumption in the LEAP Study: feasibility, growth, and nutrition. *J. Allergy Clin. Immunol.* 138 1108–1118. 10.1016/j.jaci.2016.04.016 27297994 PMC5056823

[B21] Flint H. J. Scott K. P. Louis P. Duncan S. H. (2012). The role of the gut microbiota in nutrition and health. *Nat. Rev. Gastroenterol. Hepatol.* 9 577–589. 10.1038/nrgastro.2012.156 22945443

[B22] Forsythe S. J. (2016). “Emerging foodborne enteric bacterial pathogens,” in *Encyclopedia of Food and Health*, eds caballero B. Finglas P. Toldra F. (Oxford: Acadamic Press), 487–497. 10.1016/B978-0-12-384947-2.00248-8

[B23] Gorelik L. Constant S. Flavell R. A. (2002). Mechanism of transforming growth factor beta-induced inhibition of T helper type 1 differentiation. *J. Exp. Med.* 195 1499–1505. 10.1084/jem.20012076 12045248 PMC2193549

[B24] Gorelik L. Flavell R. A. (2002). Transforming growth factor-beta in T-cell biology. *Nat. Rev. Immunol.* 2 46–53. 10.1038/nri704 11905837

[B25] Hardy H. Harris J. Lyon E. Beal J. Foey A. D. (2013). Probiotics, prebiotics and immunomodulation of gut mucosal defences: homeostasis and immunopathology. *Nutrients* 5 1869–1912. 10.3390/nu5061869 23760057 PMC3725482

[B26] Hasler C. M. (2002). Functional foods: benefits, concerns and challenges-a position paper from the american council on science and health. *J. Nutr.* 132 3772–3781. 10.1002/mus.20330 12468622

[B27] Hayes S. R. Vargas A. J. (2016). Probiotics for the prevention of pediatric antibiotic-associated diarrhea. *Explore* 12 463–466. 10.1016/j.explore.2016.08.015 27688016 PMC5140692

[B28] Hoffmann S. Batz M. Morris J. G. (2012). Annual cost of illness and quality-adjusted life year losses in the United States fue to 14 foodborne pathogens. *J. Food Prot.* 75 1292–1302. 10.4315/0362-028X.JFP-11-417 22980013

[B29] Hontecillas R. Wannemeulher M. J. Zimmerman D. R. Hutto D. L. Wilson J. H. Ahn D. U. (2002). Nutritional regulation of porcine bacterial-induced colitis by conjugated linoleic acid. *J. Nutr.* 132 2019–2027. 12097686 10.1093/jn/132.7.2019

[B30] Hsieh J. Williams P. Rafei M. Birman E. Cuerquis J. Yuan S. (2012). Inducible IL10 suppressor B cells inhibit CNS inflammation and T helper 17 polarization. *Mol. Ther.* 20 1767–1777. 10.1038/mt.2012.127 22760541 PMC3437591

[B31] Huang J. Y. Henao O. L. Griffin P. M. Vugia D. J. Cronquist A. B. Hurd S. (2016). Infection with pathogens transmitted commonly through food and the effect of increasing use of culture-independent diagnostic tests on surveillance — foodborne diseases active surveillance network, 10 U.S. Sites, 2012–2015. *MMWR. Morb. Mortal. Wkly. Rep.* 65 368–371. 10.15585/mmwr.mm6514a2 27077946

[B32] Kidd P. (2003). Th1/Th2 balance: the hypothesis, its limitations, and implications for health and disease. *Altern. Med. Rev.* 8 223–246. 12946237

[B33] Kihm D. J. Leyer G. J. An G. H. Johnson E. A. (1994). Sensitization of heat-treated Listeria monocytogenes to added lysozyme in milk. *Appl. Environ. Microbiol.* 60 3854–3861. 7986052 10.1128/aem.60.10.3854-3861.1994 PMC201895

[B34] Kishino S. Ogawa J. Yokozeki K. Shimizu S. (2011). Linoleic acid Isomerase in *Lactobacillus plantarum* AKU1009a proved to be a multi-component enzyme system requiring oxidoreduction cofactors. *Biosci. Biotechnol. Biochem.* 75 318–322. 10.1271/bbb.100699 21307591

[B35] Lee H. Y. Park J. H. Seok S. H. Baek M. W. Kim D. J. Lee K. E. (2006). Human originated bacteria, *Lactobacillus rhamnosus* PL60, produce conjugated linoleic acid and show anti-obesity effects in diet-induced obese mice. *Biochim. Biophys. Acta Mol. Cell Biol. Lipids* 1761 736–744. 10.1016/j.bbalip.2006.05.007 16807088

[B36] Livak K. J. Schmittgen T. D. (2001). Analysis of relative gene expression data using real-time quantitative PCR and the 2-delta-delta-CT Method. *Methods* 25 402–408. 10.1006/meth.2001.1262 11846609

[B37] Louis P. Hold G. L. Flint H. J. (2014). The gut microbiota, bacterial metabolites and colorectal cancer. *Nat. Rev. Microbiol.* 12 661–672. 10.1038/nrmicro3344 25198138

[B38] Marcobal A. Kashyap P. C. Nelson T. A. Aronov P. A. Donia M. S. Spormann A. (2013). A metabolomic view of how the human gut microbiota impacts the host metabolome using humanized and gnotobiotic mice. *ISME J.* 7 1933–1943. 10.1038/ismej.2013.89 23739052 PMC3965317

[B39] Matsuo Y. Miyoshi Y. Okada S. Satoh E. (2012). Receptor-like molecules on human intestinal epithelial cells interact with an adhesion factor from *Lactobacillus reuteri*. *Biosci. Microbiota Food Health* 31 93–102. 10.12938/bmfh.31.93 24936355 PMC4034283

[B40] Meraz-Torres L. S. Hernandez-Sanchez H. (2012). Conjugated linoleic acid in dairy products: a review. *Am. J. Food Technol.* 7 176–179. 10.3923/ajft.2012.176.179

[B41] Monteleone I. Pallone F. Monteleone G. (2009). Interleukin-23 and Th17 cells in the control of gut inflammation. *Mediators Inflamm.* 2009:297645. 10.1155/2009/297645 19503799 PMC2688649

[B42] Mor-Mur M. Yuste J. (2010). Emerging bacterial pathogens in meat and poultry: an overview. *Food Bioprocess Technol.* 3 24–35. 10.1007/s11947-009-0189-8

[B43] Nakamura S. Kuda T. An C. Kanno T. Takahashi H. Kimura B. (2012). Inhibitory effects of *Leuconostoc mesenteroides* 1RM3 isolated from narezushi, a fermented fish with rice, on Listeria monocytogenes infection to Caco-2 cells and A/J mice. *Anaerobe* 18 19–24. 10.1016/j.anaerobe.2011.11.006 22193553

[B44] O’connell K. J. Motherway M. O. C. Hennessey A. A. Brodhun F. Ross R. P. Feussner I. (2013). Identification and characterization of an oleate hydratase-encoding gene from Bifidobacterium breve. *Bioengineered* 4 313–321. 10.4161/bioe.24159 23851389 PMC3813531

[B45] O’Flaherty S. J. Klaenhammer T. R. (2010). Functional and phenotypic characterization of a protein from *Lactobacillus acidophilus* involved in cell morphology, stress tolerance and adherence to intestinal cells. *Microbiology* 156 3360–3367. 10.1099/mic.0.043158-0 20829293

[B46] O’Shea E. F. Cotter P. D. Stanton C. Ross R. P. Hill C. (2012). Production of bioactive substances by intestinal bacteria as a basis for explaining probiotic mechanisms: Bacteriocins and conjugated linoleic acid. *Int. J. Food Microbiol.* 152 189–205. 10.1016/j.ijfoodmicro.2011.05.025 21742394

[B47] Pandey K. R. Naik S. R. Vakil B. V. (2015). Probiotics, prebiotics and synbiotics- a review. *J. Food Sci. Technol.* 52 7577–7587. 10.1007/s13197-015-1921-1 26604335 PMC4648921

[B48] Pandit A. Anand S. Kalscheur K. Hassan A. (2012). Production of conjugated linoleic acid by lactic acid bacteria in milk without any additional substrate. *Int. J. Dairy Technol.* 65 603–608. 10.1111/j.1471-0307.2012.00870.x

[B49] Peng M. Aryal U. Cooper B. Biswas D. (2015a). Metabolites produced during the growth of probiotics in cocoa supplementation and the limited role of cocoa in host-enteric bacterial pathogen interactions. *Food Control* 53 124–133. 10.1016/j.foodcont.2015.01.014

[B50] Peng M. Bitsko E. Biswas D. (2015b). Functional properties of peanut fractions on the growth of probiotics and foodborne bacterial pathogens. *J. Food Sci.* 80 M635–M641. 10.1111/1750-3841.12785 25627431

[B51] Peng M. Reichmann G. Biswas D. (2015c). *Lactobacillus casei* and its byproducts alter the virulence factors of foodborne bacterial pathogens. *J. Funct. Foods* 15 418–428. 10.1016/j.jff.2015.03.055

[B52] Peng M. Biswas D. (2017). Short chain and polyunsaturated fatty acids in host gut health and foodborne bacterial pathogen inhibition. *Crit. Rev. Food Sci. Nutr.* 57 3987–4002. 10.1080/10408398.2016.1203286 27438132

[B53] Peng M. Patel P. Vinod N. Cassandra B. Michael C. Debabrata B. (2018a). “Feasible options to control colonization of enteric pathogens with designed synbiotics,” in *Dietary Interventions in Gastrointestinal Diseases*, eds Watson R. Preedy V. (Amsterdam: ELSEVIER).

[B54] Peng M. Salaheen S. Buchanan R. L. Biswas D. (2018b). Alterations of *Salmonella typhimurium* antibiotic resistance under environmental pressure. *Appl. Environ. Microbiol.* 84:e01173-18. 10.1128/AEM.01173-18AEM.01173-18, 30054356 PMC6146977

[B55] Peng M. Salaheen S. Almario J. A. Tesfaye B. Buchanan R. Biswas D. (2016). Prevalence and antibiotic resistance pattern of *Salmonella serovars* in integrated crop-livestock farms and their products sold in local markets. *Environ. Microbiol.* 18 1654–1665. 10.1111/1462-2920.13265 26914740

[B56] Peng M. Salaheen S. Biswas D. (2014). “Animal health: global antibiotic issues,” in *Encyclopedia of Agriculture and Food Systems*, ed. Van Alfen N. K. (Amsterdam: Elsevier Ltd.), 346–357. 10.1016/B978-0-444-52512-3.00187-X

[B57] Peng M. Zhao X. Biswas D. (2017). Polyphenols and tri-terpenoids from Olea europaea L. in alleviation of enteric pathogen infections through limiting bacterial virulence and attenuating inflammation. *J. Funct. Foods* 36 132–143. 10.1016/j.jff.2017.06.059

[B58] Renner L. D. Weibel D. B. (2011). Physicochemical regulation of biofilm formation. *MRS Bull.* 36 347–355. 10.1557/mrs.2011.65 22125358 PMC3224470

[B59] Rizos E. C. Ntzani E. E. Bika E. Kostapanos M. S. Elisaf M. S. (2012). Association between omega-3 fatty acid supplementation and risk of major cardiovascular disease events: a systematic review and meta-analysis. *JAMA* 308 1024–1033. 10.1001/2012.jama.11374 22968891

[B60] Romagnani S. (1999). Th1/Th2 cells. *Inflamm. Bowel Dis.* 5 285–294. 10.1097/00054725-199911000-00009 10579123

[B61] Salaheen S. Jaiswal E. Joo J. Peng M. Ho R. OConnor D. (2016a). Bioactive extracts from berry byproducts on the pathogenicity of *Salmonella Typhimurium*. *Int. J. Food Microbiol.* 237 128–135. 10.1016/j.ijfoodmicro.2016.08.027 27565525

[B62] Salaheen S. Peng M. Biswas D. (2016b). Ecological dynamics of campylobacter in integrated mixed crop–livestock farms and its prevalence and survival ability in post-harvest products. *Zoonoses Public Health* 63 641–650. 10.1111/zph.12279 27178350

[B63] Salaheen S. Peng M. Biswas D. (2015). *Replacement of Conventional Antimicrobials and Preservatives in Food Production to Improve Consumer Safety and Enhance Health Benefits.* Abingdon: Taylor and Francis.

[B64] Salaheen S. Peng M. Joo J. Teramoto H. Biswas D. (2017). Eradication and sensitization of methicillin resistant Staphylococcus aureus to methicillin with bioactive extracts of berry pomace. *Front. Microbiol.* 8:253. 10.3389/fmicb.2017.00253 28270804 PMC5319404

[B65] Salaheen S. White B. Bequette B. J. Biswas D. (2014). Peanut fractions boost the growth of Lactobacillus casei that alters the interactions between *Campylobacter jejuni* and host epithelial cells. *Food Res. Int.* 62 1141–1146. 10.1016/j.foodres.2014.05.061

[B66] Serini S. Piccioni E. Merendino N. Calviello G. (2009). Dietary polyunsaturated fatty acids as inducers of apoptosis: implications for cancer. *Apoptosis* 14 135–152. 10.1007/s10495-008-0298-2 19130233

[B67] Stanton C. Ross R. P. Fitzgerald G. F. Van Sinderen D. (2005). Fermented functional foods based on probiotics and their biogenic metabolites. *Curr. Opin. Biotechnol.* 16 198–203. 10.1016/j.copbio.2005.02.008 15831387

[B68] Tabashsum Z. Peng M. Salaheen S. Comis C. Biswas D. (2018). Competitive elimination and virulence property alteration of *Campylobacter jejuni* by genetically engineered *Lactobacillus casei*. *Food Control* 85 283–291. 10.1016/J.FOODCONT.2017.10.010

[B69] Tricon S. Burdge G. C. Kew S. Banerjee T. Russell J. J. Grimble R. F. (2004). Effects of cis-9,trans-11 and trans-10,cis-12 conjugated linoleic acid on immune cell function in healthy humans. *Am. J. Clin. Nutr.* 80 1626–1633. 15585778 10.1093/ajcn/80.6.1626

[B70] Turnock L. Cook M. Steinberg H. Czuprynski C. (2001). Dietary supplementation with conjugated linoleic acid does not alter the resistance of mice to *Listeria monocytogenes* infection. *Lipids* 36 135–138. 11269693 10.1007/s11745-001-0699-3

[B71] Van Nieuwenhove C. P. Cano P. G. Pérez-Chaia A. B. González S. N. (2011). Effect of functional buffalo cheese on fatty acid profile and oxidative status of liver and intestine of mice. *J. Med. Food* 14 420–427. 10.1089/jmf.2010.0061 21370968

[B72] Viswanathan V. K. Hodges K. Hecht G. (2009). Enteric infection meets intestinal function: how bacterial pathogens cause diarrhoea. *Nat. Rev. Microbiol.* 7 110–119. 10.1038/nrmicro2053 19116615 PMC3326399

[B73] Volkov A. Liavonchanka A. Kamneva O. Fiedler T. Goebel C. Kreikemeyer B. (2010). Myosin cross-reactive antigen of *Streptococcus pyogenes* M49 encodes a fatty acid double bond hydratase that plays a role in oleic acid detoxification and bacterial virulence. *J. Biol. Chem.* 285 10353–10361. 10.1074/jbc.M109.081851 20145247 PMC2856241

[B74] Vu B. Chen M. Crawford R. J. Ivanova E. P. (2009). Bacterial extracellular polysaccharides involved in biofilm formation. *Molecules* 14 2535–2554. 10.3390/molecules14072535 19633622 PMC6254922

[B75] Vyas U. Ranganathan N. (2012). Probiotics, prebiotics, and synbiotics: gut and beyond. *Gastroenterol. Res. Pract.* 2012: 872716. 10.1155/2012/872716 23049548 PMC3459241

[B76] Yang B. Chen H. Gu Z. Tian F. Ross R. P. Stanton C. (2014). Synthesis of conjugated linoleic acid by the linoleate isomerase complex in food-derived lactobacilli. *J. Appl. Microbiol.* 117 430–439. 10.1111/jam.12524 24750362 PMC4306591

[B77] Yang B. Chen H. Stanton C. Ross R. P. Zhang H. Chen Y. Q. (2015). Review of the roles of conjugated linoleic acid in health and disease. *J. Funct. Foods* 15 314–325. 10.1016/j.jff.2015.03.050

[B78] Yang B. Gao H. Stanton C. Ross R. P. Zhang H. Chen Y. Q. (2017). Bacterial conjugated linoleic acid production and their applications. *Prog. Lipid Res.* 68 26–36. 10.1016/j.plipres.2017.09.002 28889933

